# Hepatic focal nodular hyperplasia after liver transplantation: case report and review of literature

**DOI:** 10.1007/s12328-025-02180-5

**Published:** 2025-07-18

**Authors:** Kenichi Nakamura, Yoshinori Ozono, Satoru Hasuike, Hiroshi Hatada, Naomi Uchiyama, Yuri Komaki, Hisayoshi Iwakiri, Kenji Nagata, Yoshiko Umekita, Shinichi Aishima, Hiroshi Kawakami

**Affiliations:** 1 Division of Gastroenterology and Hepatology, Department of Internal Medicine, Faculty of Medicine, University of Miyazaki, 5200 Kihara, Kiyotake, Miyazaki, 889-1692 Japan; 2 Section of Oncopathology and Morphological Pathology, Department of Pathology, Faculty of Medicine, University of Miyazaki, Miyazaki, Japan; 3 Department of Scientific Pathology Graduate School of Medical Sciences, Kyushu University, Fukuoka, Japan

**Keywords:** Focal nodular, Hyperplasia, Hepatocellular carcinoma, Liver transplantation

## Abstract

Patients with decompensated cirrhosis complicated by hepatocellular carcinoma (HCC) or those who have undergone liver transplantation following liver failure after HCC treatment should continue to receive post-transplant surveillance. Any new liver tumor must be carefully evaluated to determine whether it is a recurrence of HCC. Focal nodular hyperplasia (FNH), the second most common benign hepatic tumor, is believed to result from the hyperplastic response of hepatocytes to pre-existing vascular malformation. Here, we report a case of hepatic FNH two years after living-donor liver transplantation in a patient who experienced liver failure following treatment for HCC. Given the rarity of hepatic FNH after liver transplantation, we present this case along with a review of the literature.

## Introduction

Focal nodular hyperplasia (FNH) is a hyperplastic hepatocellular lesion associated with vascular malformation [1]. According to the World Health Organization (WHO) classification, FNH is considered a tumor-forming non-neoplastic lesion of hepatocellular origin, along with hepatocellular adenoma (HCA) [2]. FNH is the second most frequent benign hepatic tumor after hemangioma, with an incidence of 0.3–3% and a frequency of 0.8% in autopsy cases. It is more common in women aged 20–50 [3–5]. On contrast-enhanced computed tomography (CECT), FNH is hyperattenuating in the arterial phase and almost isoattenuating with the surrounding liver in the portal venous and equilibrium phases. In gadoxetic acid-enhanced magnetic resonance imaging (EOB-MRI), FNH shows hyperintensity in the arterial phase and iso- to slight hyperintensity in the portal venous and equilibrium phases [6–9]. Some FNHs also exhibit ring-like enhancement in the hepatobiliary phase of EOB-MRI, a characteristic finding of FNH [10]. Although long-term observation reports of FNH are few, it rarely increases in size [11], and follow-up is considered acceptable once the diagnosis is confirmed [12, 13]. Treatment of FNH is considered when symptoms such as abdominal pain appear, the tumor increases in size, or it becomes difficult to distinguish from a malignant tumor.

Radical treatment of FNH involves hepatic resection, but transarterial chemoembolization (TACE) and percutaneous radiofrequency ablation are also options [14]. We encountered a case of hepatic FNH two years after liver transplantation, a rare occurrence with few reported cases. Since FNH, like HCC, appears as a hypervascular tumor on imaging, distinguishing between the two is crucial. This differentiation will be discussed in a literature review.

## Case presentation

A 72-year-old man was diagnosed with alcoholic cirrhosis in 2012. He developed multiple HCCs in January 2018 and underwent TACE, and proton beam therapy in April 2018. The imaging studies showed no intrahepatic recurrence of HCC; however, he developed liver failure and underwent living-donor liver transplantation in February 2020. Four nodules with well-to-moderately differentiated HCCs were found in the excised liver. After the liver transplantation, he was administered immunosuppressant, such as tacrolimus and mycophenolate. The patient was referred to our hospital in March 2020 for post-transplant follow-up.

**Fig. 1:**
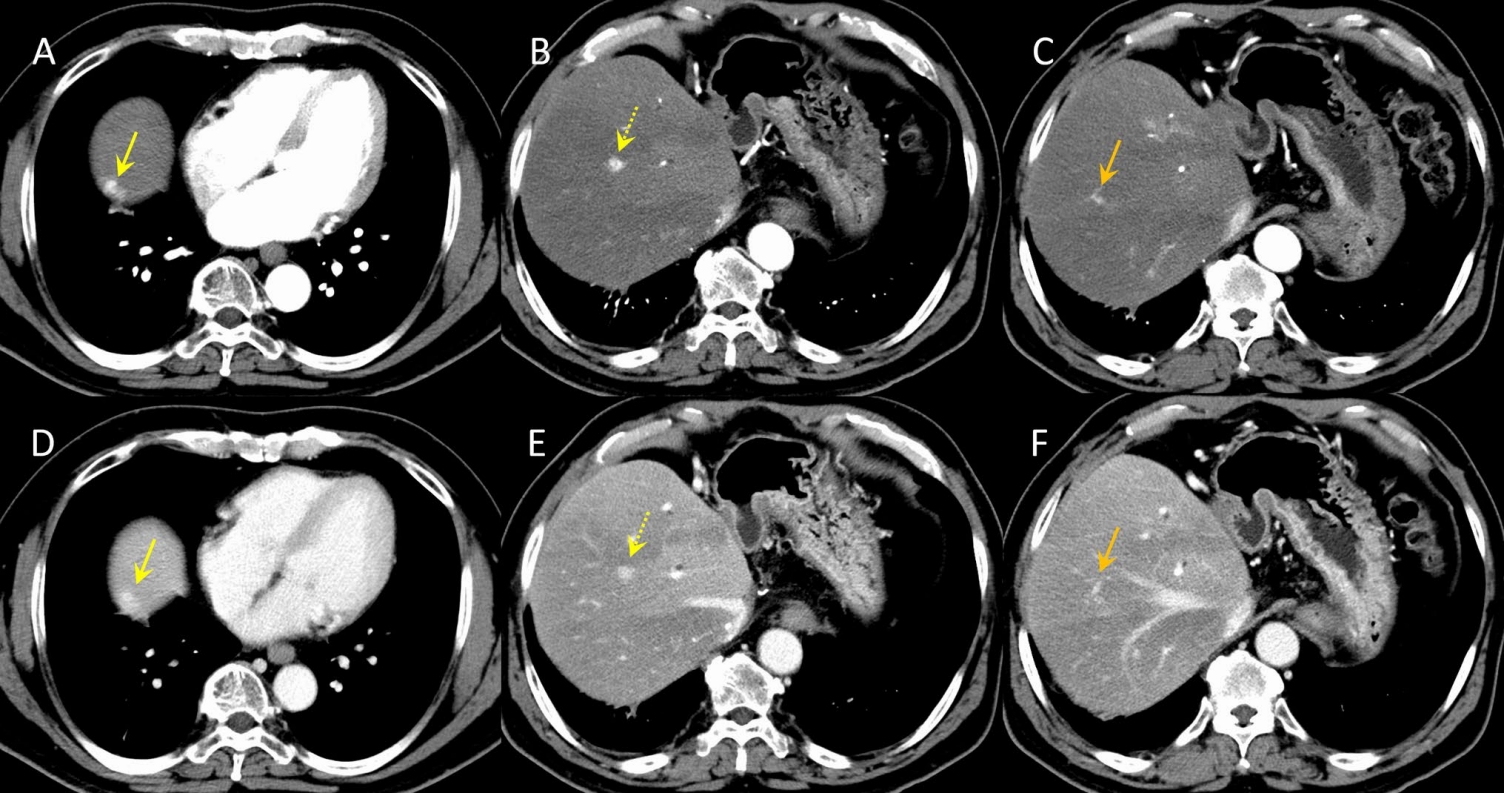
Contrast-enhanced computed tomography images of focal nodular hyperplasia one year after liver transplantation. Three lesions were hyperattenuating in the arterial phase (**A**: dome, yellow arrow, **B**: S8, dotted yellow arrow, **C**: S8/7, orange arrow). These lesions remained hyperattenuating in the portal venous phase (**D**: dome, yellow arrow, **E**: S8, dotted yellow arrow, **F**: S8/7, orange arrow)

**Fig. 2:**
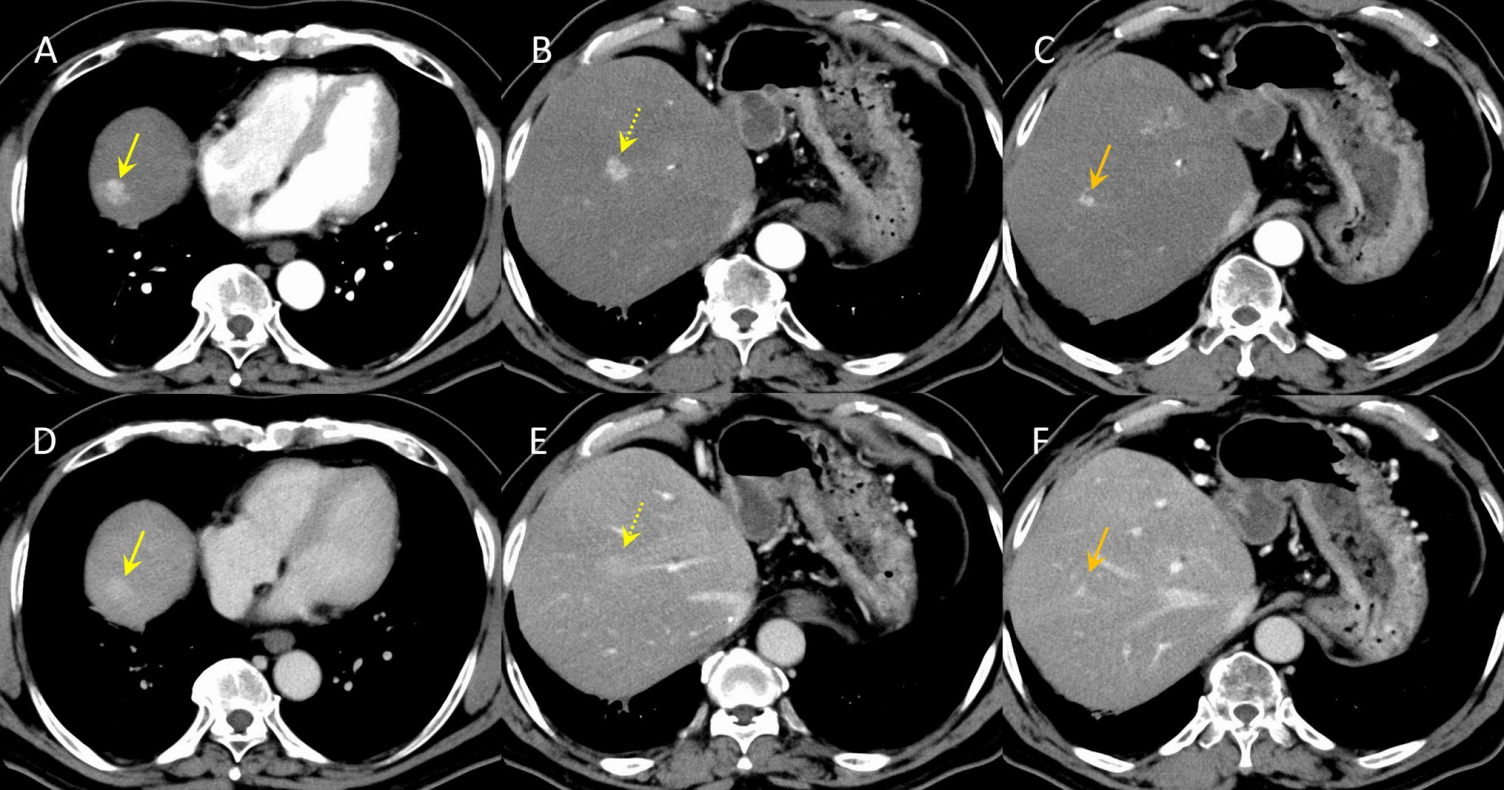
Contrast-enhanced computed tomography images of focal nodular hyperplasia two years after liver transplantation. Three lesions were hyperattenuating in the arterial phase (**A**: dome, yellow arrow, **B**: S8, dotted yellow arrow, **C**: S8/7, orange arrow). These lesions remained hyperattenuating in the portal venous phase (**D**: dome, yellow arrow, **E**: S8, dotted yellow arrow, **F**: S8/7, orange arrow)

**Fig. 3:**
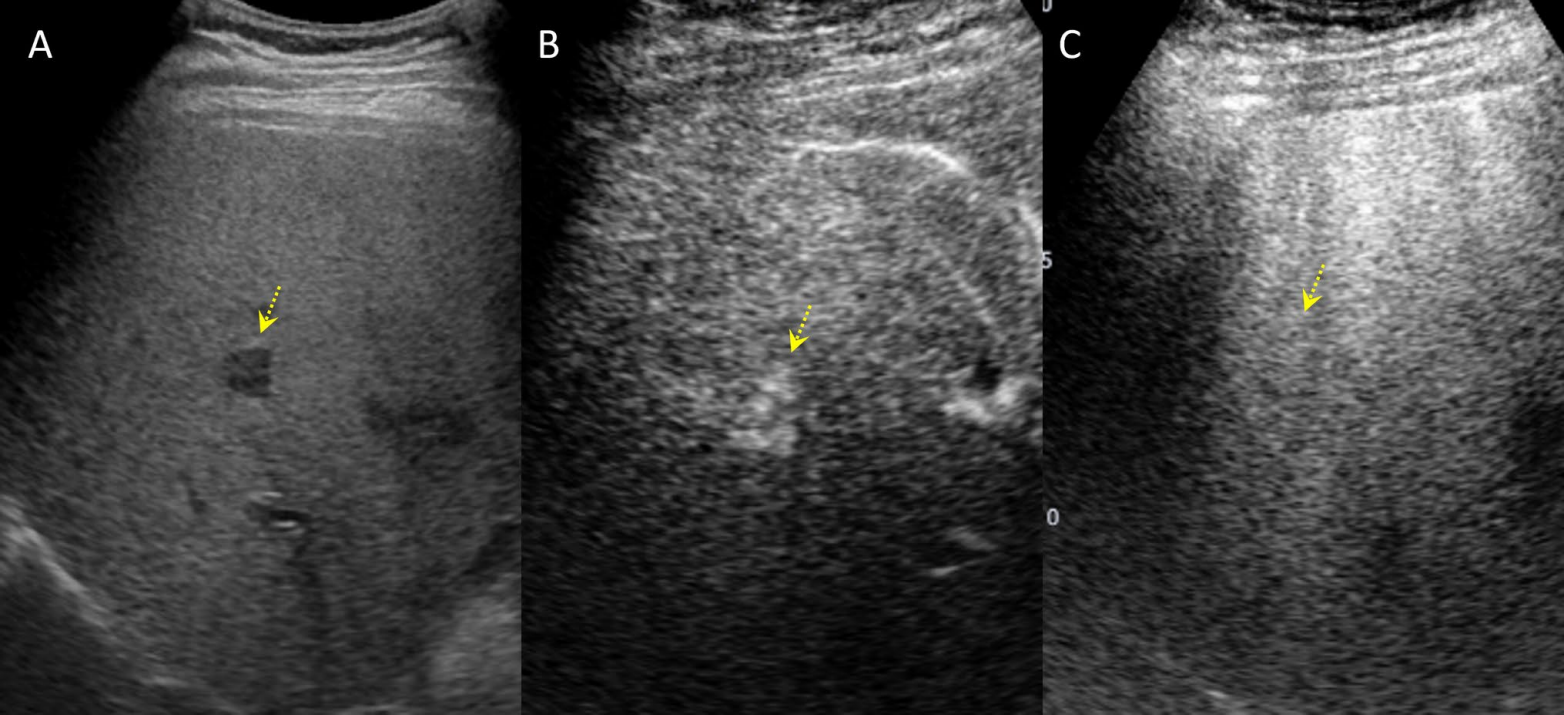
Contrast-enhanced ultrasound images of focal nodular hyperplasia (S8) (dotted yellow arrows) two years after liver transplantation. **A** The liver nodule was depicted as a 13 mm hypoechoic area in B-mode. **B** The liver nodule was hyperattenuating in the arterial phase. **C** There was no defect in the Kupffer phase

**Fig. 4:**
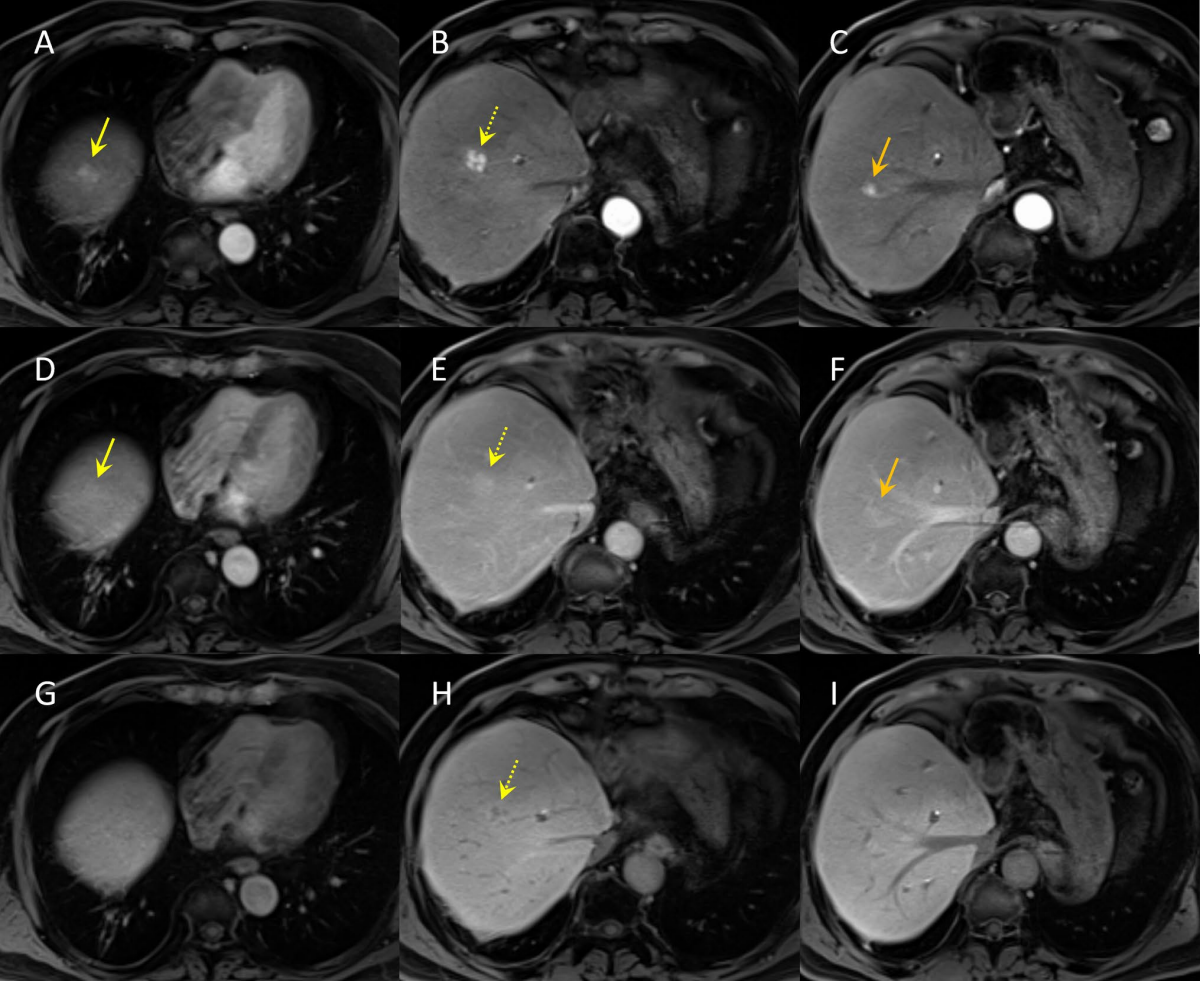
Gadoxetic acid-enhanced magnetic resonance imaging of focal nodular hyperplasia three years after liver transplantation. Three lesions were hyperintense in the arterial phase (**A**: dome, yellow arrow, **B**: S8, dotted yellow arrow, **C**: S8/7, orange arrow). These lesions remained hyperintense in the portal venous phase (**D**: dome, yellow arrow, **E**: S8, dotted yellow arrow, **F**: S8/7, orange arrow). Dome and S8/7 lesions were isointense, but S8 lesion showed hypointensity inside and hyperintensity outside in the hepatobiliary phase (**G**: dome, yellow arrow, **H**: S8, dotted yellow arrow, **I**: S8/7, orange arrow)

**Fig. 5:**
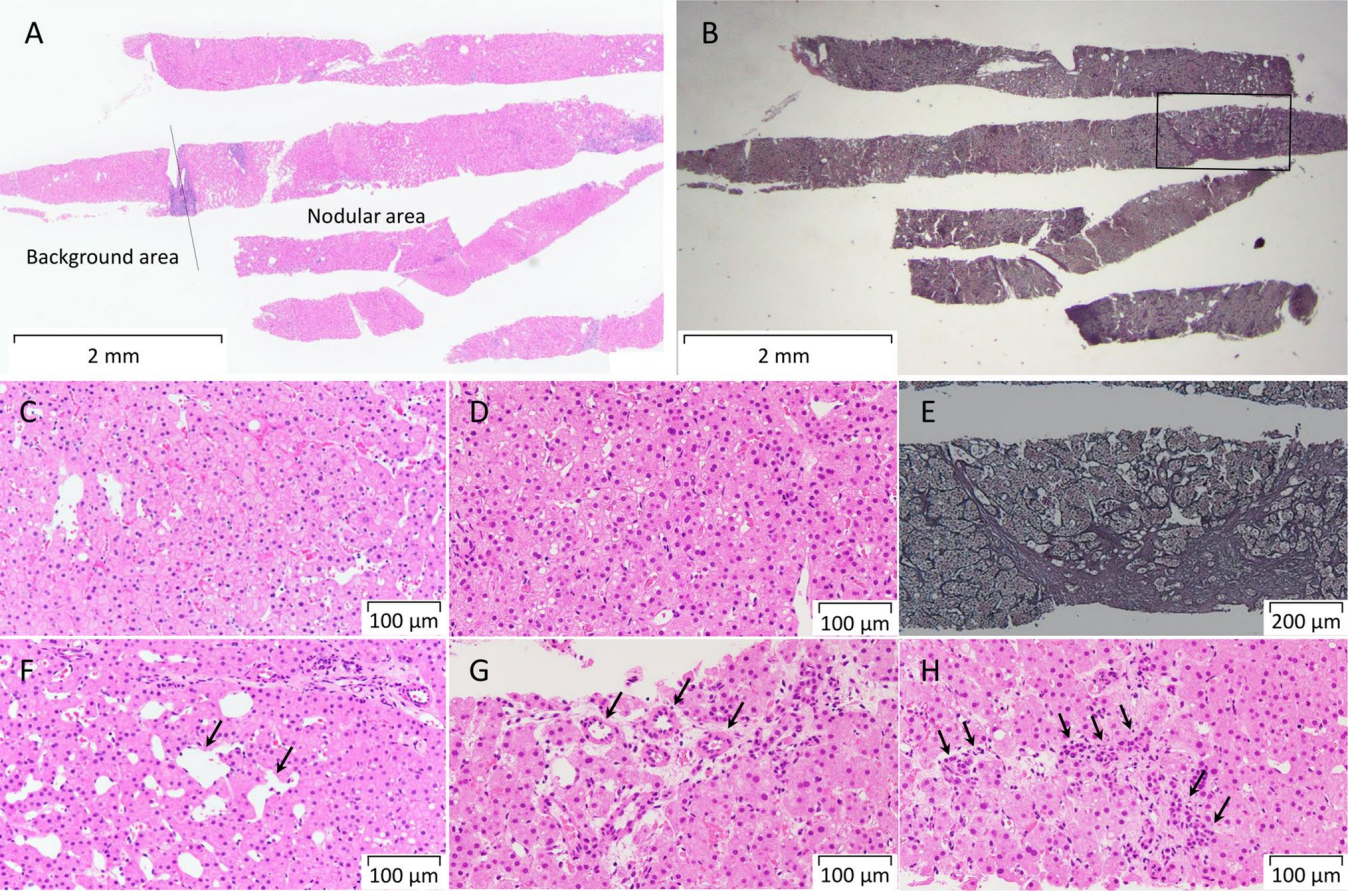
H&E and Silver stain of focal nodular hyperplasia. **A** Most of the tissue is nodule with a small amount of background liver visible to the left of the line. **B** Silver Stain; There is a small amount of background liver, no fibrosis is observed there. Fibrosis is observed in the tumor area enclosed by the square. **C** Enlarged image of Background area. **D** Enlarged image of Nodular area; increased hepatocyte density, but no cellular atypia. **E** Silver Stain; Enlarged image of the area enclosed by the square in B, fibrosis within the tumor, but no central scar was observed. The tumor contains dilated sinusoids (**F**, black arrow), abnormal muscular vessels (**G**, black arrow), and ductal reactions (**H**, black arrow), which are characteristic findings of FNH

**Fig. 6:**
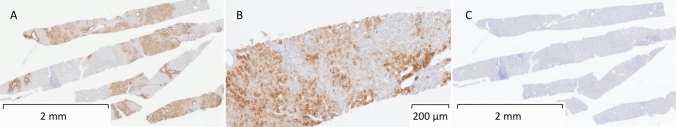
Immunostaining of focal nodular hyperplasia. **A** Glutamine synthetase, positive in a “map-like” pattern. This is a characteristic finding of FNH. **B** Glutamine synthetase, enlarged image. **C** Glypican 3, a specific antigen for hepatocellular carcinoma, was negative

In April of the same year, the patient developed cholangitis due to stenosis of the bile duct anastomosis, and transpapillary stent placement was performed. Subsequently, the stents were replaced routinely. One year after the liver transplantation, CECT revealed three hyperattenuating lesions (maximum diameter 10 mm) in the right lobe of the liver that did not show wash-out in the portal venous and equilibrium phases (Fig. 1). Since no obvious nodules were detected on ultrasonography (US), the lesion was initially thought to be an arterioportal shunt and was followed up. Two years after the liver transplantation, CECT showed that these hyperattenuating lesions had increased in size (Fig. 2). B-mode US revealed one hypoechoic nodule, approximately 10 mm in diameter, in the right lobe of the liver (Fig. 3A). We considered it to be a nodule in S8. Contrast-enhanced US (CEUS) with Sonazoid showed that the nodule was hyperattenuating in the early vascular phase with no defect in the Kupffer phase (Fig. 3B, C). CEUS did not show a spoke-wheel pattern, which indicate blood flow from the center of the lesion to the periphery. Since there was no increase in tumor markers (AFP, PIVKA-II, CEA, and CA19-9), FNH and HCA were suspected rather than HCC recurrence, and the patient was followed up. EOB-MRI performed 3 years after liver transplantation showed that these lesions ware hyperintense in the arterial phase, with no wash-out in the portal venous and equilibrium phases, similar to CECT findings. However, the S8 lesion showed hypointensity inside and hyperintensity outside in the hepatobiliary phase (Fig. 4). No central scar was noted within the lesion. Therefore, HCC could not be ruled out and a liver tumor biopsy was performed in July 2023. Histopathology showed areas with a slightly increased number of small hepatocytes with a high nuclear-to-cytoplasmic ratio, an increased number of bile ductules, small muscular vessels, and dilated sinusoids, but no cell atypia. Although the central scar was not evident, some areas showed mild fibrosis with inflammatory cell infiltration and ductular reaction (Fig. 5). No evident thrombi or necrotic areas were observed. Immunostaining showed the tumor was positive for glutamine synthetase in a “map-like” pattern. Serum amyloid A was negative, whereas C-reactive protein was detected in some areas. Based on these findings, the hepatic tumor was diagnosed as an FNH (Fig. 6). As of July 2024, the tumor size remained unchanged.

## Discussion

There have been only five reports of hepatic FNH occurring after liver transplantation [15, 16]. The age at FNH occurrence ranged from 2 to 62 years, with four patients being male and one female (Table 1). The time from liver transplantation to FNH development ranged from 15 to 118 months, and the mass diameter at diagnosis ranged from 1.3 to 6.7 cm. Two of the five cases had two FNHs (Table 1). The causes of liver transplantation included biliary atresia, hepatitis B, hepatitis C, and HCC. At diagnosis, the background liver fibrosis included cirrhosis in one case and chronic hepatitis in three cases (one case not mentioned). Pathologically, this case showed a mild fatty liver and mild inflammatory cell infiltration in the portal area, but no fibrosis.

**Table 1:** Clinical features of cases of focal nodular hyperplasia after liver transplantation

Case	Age	Sex	Original disease	Interval between LT and Dx of FNH (months)	Follow-up (Months)	Specimen collection method	Location	Size (cm)	Central scar	Background liver pathology	Vascular comorbidities
1 [15]	2	M	Biliary atresia	18	8	Needle core biopsy Followed by wedge resection None (identified by imaging)	Left lobe	1.7	Yes	Mild, nonspecific portal and lobular hepatitis with a moderate degree of microvesicular steatosis; no fibrosis	Living related donor transplantation
							Right	1.3	No		
2 [15]	12	F	Biliary atresia	118	70	Needle core biopsy	Dome	4.5	No	Portal vein thrombosis with sclerosing Portal venopathy; bridging fibrosis and cirrhosis	History of portal vein thrombosis
	17			188	None	Autopsy	Left lobe	1.9	Yes		
3 [15]	52	M	Hepatitis B and C, Alcoholic	105	6	Needle core biopsy	Right	6.7	No	Chronic hepatitis with mild activity and focal periportal fibrosis	History of portal vein thrombosis
4 [15]	63	M	Hepatitis	15	None	Autopsy	Right	2	No	Chronic hepatitis with mild activity and periportal fibrosis	None identifled
5 [16]	64	M	NAFLD/HCC	60	None	Needle core biopsy	Right	2.6	No	ND	History of portal vein thrombosis
6 (present case)	67	M	Alcoholic/HCC	41	12	Needle core biopsy	Right	2	No	Mild portal hepatitis with mild degree of microvesicular steatosis; no fibrosis	Living related donor transplantation

*LT* liver transplantation, *Dx* diagnosis, *FNH* focal nodular hyperplasia, *NAFLD* nonalcoholic fatty liver disease, *HCC* hepatocellular carcinoma, *ND* not described

Transplant-induced vascular changes, such as intraoperative vascular manipulation and vascular anastomosis, are thought to cause hepatic FNH after liver transplantation [15, 16]. Wanless et al. reported that impaired liver perfusion releases platelet-derived growth factors from hepatocytes, which can cause hyperplasia of hepatocytes [3]. They also noted that a major abnormality of FNH is an increase in regional arterial blood flow [3]. Kumagai et al. concluded that FNH begins with thrombosis of the hepatic artery or portal vein, leading to local hepatic ischemia or necrosis, followed by reopening of the hepatic artery and transient tissue hyperperfusion, which results in nodule formation [17].

Three of the five previously reported cases of hepatic FNH occurring after liver transplantation had a history of portal vein thrombosis (PVT), which is considered a contributing factor to FNH development [15, 16]. However, there was no obvious history of PVT in this case. Ra et al. noted that the occurrence of FNH after living donor liver transplantation is plausible [15]. This is likely because living-donor liver transplantation involves more extensive manipulation of liver vessels than brain-dead donor liver transplantation, making it more susceptible to thrombosis [18]. Additionally, post-liver transplant patients face an increased risk of coagulation abnormalities, rejection, and infection, which may further contribute to thrombosis [19]. In this case, the patient experienced recurrent cholangitis associated with stenosis of the bile duct anastomosis. A needle biopsy was performed and no obvious thrombus was found in the specimens. However, thrombus formation in the small portal vein and hepatic artery may have contributed to the development of FNH. Furthermore, CECT performed one year after transplantation revealed a hyperattenuating lesion in the liver, which was undetectable on B-mode US but was identifiable as a tumor two years later. The liver biopsy specimens did not have enough of the background liver; therefore, it was difficult to state that immunosuppressant and rejection affected intrahepatic blood flow and development of FNH in this case.

We believe that the increase in small muscular vessels observed in this case reflects the developmental process of FNH and provides valuable insights into its progression through imaging. In this case, FNH exhibited hypointensity in the hepatobiliary phase on EOB-MRI, which was atypical. Consequently, HCA or recurrence of HCC was also considered; however, the liver biopsy results confirmed the diagnosis of FNH. There were three lesions present and only the largest was diagnosed as FNH through liver biopsy. Therefore, careful follow-up with imaging studies is necessary for the other two smaller lesions. Since FNH, like HCC, appears hypervascular on imaging, it should be considered when new lesions are detected in the liver after transplantation. Furthermore, if imaging findings are atypical for FNH, as in this case, a liver biopsy is essential to establish the correct diagnosis. FNH is potentially more prevalent in liver transplant recipients than in the general population. Due to the lack of cases described, the diagnostic potential may be overlooked by pathologists and clinicians. FNH has not been acknowledged as a potential etiology of liver nodules post-transplant and warrants consideration in the differential diagnosis of hepatic nodules in transplanted livers.

## References

[1] Kondo F. Benign nodular hepatocellular lesions caused by abnormal hepatic circulation: etiological analysis and introduction of a new concept. J Gastroenterol Hepatol. 2001;16:1319–28. 11851827 10.1046/j.1440-1746.2001.02576.x

[2] Bioulac-Sage P, Kakar S, Nault JC. Hepatocellular adenoma. In: The WHO Classification of Tumours Editorial Board, Ed. WHO Classification of Tumours: Digestive System Tumours, 5th ed. IARC Press: Lyon; 2019. pp. 224–228

[3] Wanless IR, Mawdsley C, Adams R. On the pathogenesis of focal nodular hyperplasia of the liver. Hepatology. 1985;5:1194–200. 4065824 10.1002/hep.1840050622

[4] Nguyen BN, Fléjou JF, Terris B, et al. Focal nodular hyperplasia of the liver: a comprehensive pathologic study of 305 lesions and recognition of new histologic forms. Am J surg Pathol. 1999;23:1441–54. 10584697 10.1097/00000478-199912000-00001

[5] Vilgrain V, Uzan F, Brancatelli G, et al. Prevalence of hepatic hemangioma in patients with focal nodular hyperplasia: MR imaging analysis. Radiology. 2003;229:75–9. 12944594 10.1148/radiol.2291021284

[6] Lin MC, Tsay PK, Ko SF, et al. Triphasic dynamic CT findings of 63 hepatic focal nodular hyperplasia in 46 patients: correlation with size and pathological findings. Abdom Imaging. 2008;33:301–7. 17632749 10.1007/s00261-007-9258-5

[7] Zech CJ, Grazioli L, Breuer J, et al. Diagnostic performance and description of morphological features of focal nodular hyperplasia in Gd-EOB-DTPA-enhanced liver magnetic resonance imaging: results of a multicenter trial. Invest Radiol. 2008;43:504–11. 18580333 10.1097/RLI.0b013e3181705cd1

[8] Karam AR, Shankar S, Surapaneni P, et al. Focal nodular hyperplasia: central scar enhancement pattern using gadoxetate disodium. J Magn Rwson Imaging. 2010;32:341–4. 10.1002/jmri.22262 20677260

[9] Grazioli L, Bondioni MP, Haradome H, et al. Hepatocellular adenoma and focal nodular hyperplasia: value of gadoxetic acid-enhanced MR imaging in differential diagnosis. Radiology. 2012;262:520–9. 22282184 10.1148/radiol.11101742

[10] Fujiwara H, Sekine S, Onaya H, et al. Ring-like enhancement of focal nodular hyperplasia with hepatobiliary-phase Gd-EOB-DTPA-enhanced magnetic resonance imaging: radiological-pathological correlation. Jpn J Radiol. 2011;29:739–43. 22009428 10.1007/s11604-011-0624-4

[11] Kuo YH, Wang JH, Lu SN, et al. Natural course of hepatic focal nodular hyperplasia: a long-term follow-up study with sonography. J Clin Ultrasound. 2009;37:132–7. 18855931 10.1002/jcu.20533

[12] Rogers JV, Mack LA, Freeny PC, et al. Hepatic focal nodular hyperplasia: angiography, CT, sonography, and scintigraphy. AJR. 1981;137:983–90. 6975026 10.2214/ajr.137.5.983

[13] Fechner RE, Roehm JO. Angiographic and pathologic correlations of hepatic focal nodular hyperplasia. Am J Surg Pathol. 1977;1:217–24. 920869 10.1097/00000478-197709000-00003

[14] Hedayati P, VanSonnenberg E, Shamos R, et al. Treatment of symptomatic focal nodular hyperplasia with percutaneous radiofrequency ablation. J Vasc Interv Radiol. 2010;21:582–5. 20138547 10.1016/j.jvir.2009.12.385

[15] Ra SH, Kaplan JB, Lassman CR. Focal nodular hyperplasia after orthotopic liver transplantation. Liver Transpl. 2010;16:98–103. 19866450 10.1002/lt.21956

[16] Gainey CS, Palmer SL, Mena E, et al. Diagnosis of focal nodular hyperplasia (FNH) after liver transplantation. Case Rep Transplant. 2020;2020:8824099. 33083085 10.1155/2020/8824099 PMC7556100

[17] Kumagai H, Masuda T, Oikawa H, et al. Focal nodular hyperplasia of the liver: direct evidence of circulatory disturbances. J Gastroenterol Hepatol. 2000;15:1344–7. 11129233

[18] Florman S, Miller CM. Live donor liver transplantation. Liver Transpl. 2006;12:499–510. 16555328 10.1002/lt.20754

[19] Demetris AJ, Nalesnik M, Randhawa P, et al. Histologic patterns of rejection and other causes of liver dysfunction. In: Busuttil RW, Klintmalm GB, editors., et al., Transplantation of the Liver. 2nd ed. Philadelphia, PA: Elsevier Saunders; 2005. p. 1073–4.

